# Supplementary figures and images for: Genesis of the αβ T-cell receptor
Source: PLoS Comput Biol. 2019 Mar 4;15(3):e1006874. doi: 10.1371/journal.pcbi.1006874 (PMC6417744; doi:10.1371/journal.pcbi.1006874)

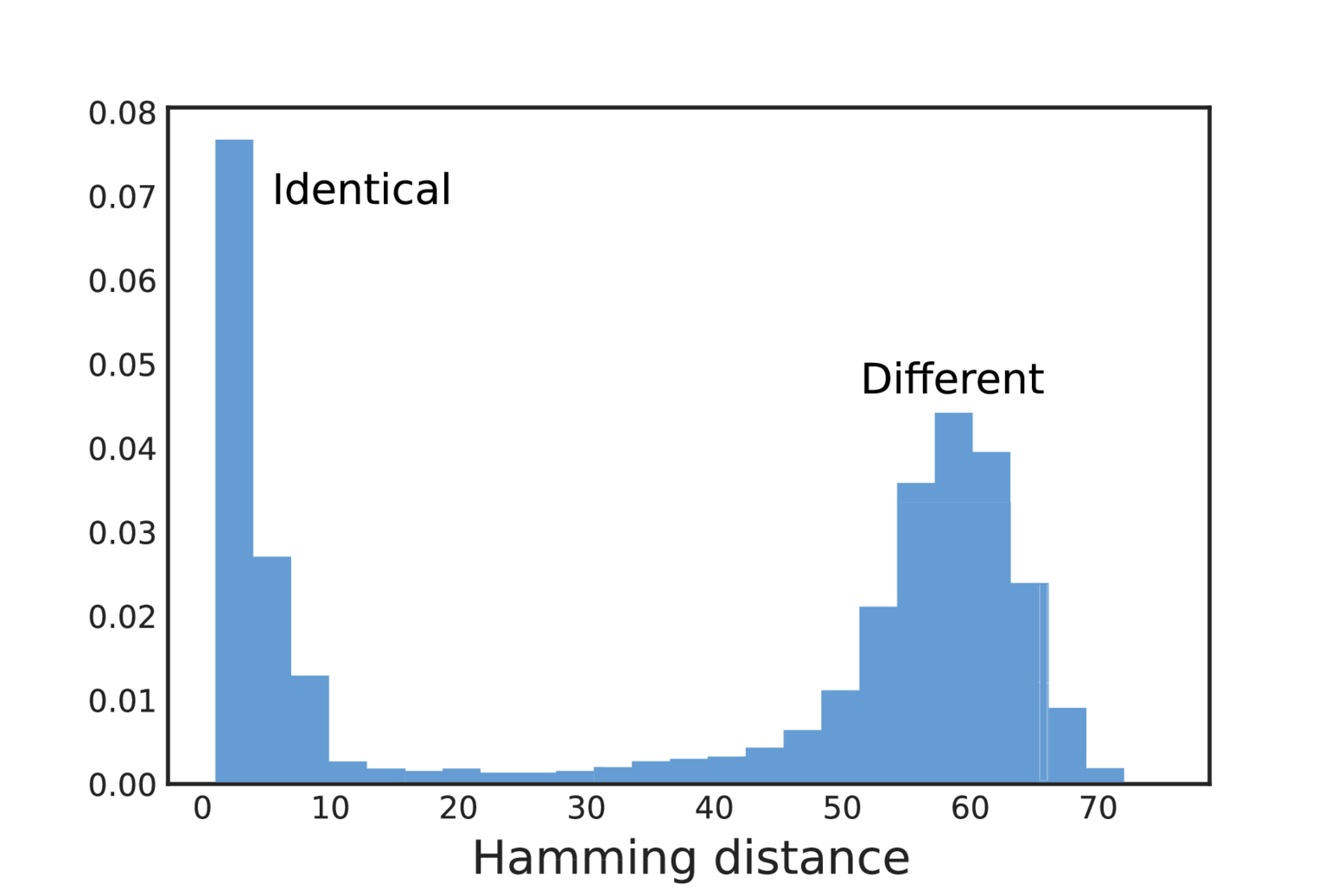

Supplement: S1 Fig — Near-identical paired sequences are in their vast majority due to sequencing error. The Hamming distance permits to separate effectively these sequences from actually different sequences extracted from the same clone. A similar behaviour is observed for TCRα chains. A threshold of 11 was chosen to exclude pairs from sequencing errors from the analysis, while retaining as many pairs as possible, including some with the same gene usage. (TIF) [file pcbi.1006874.s001.tif]

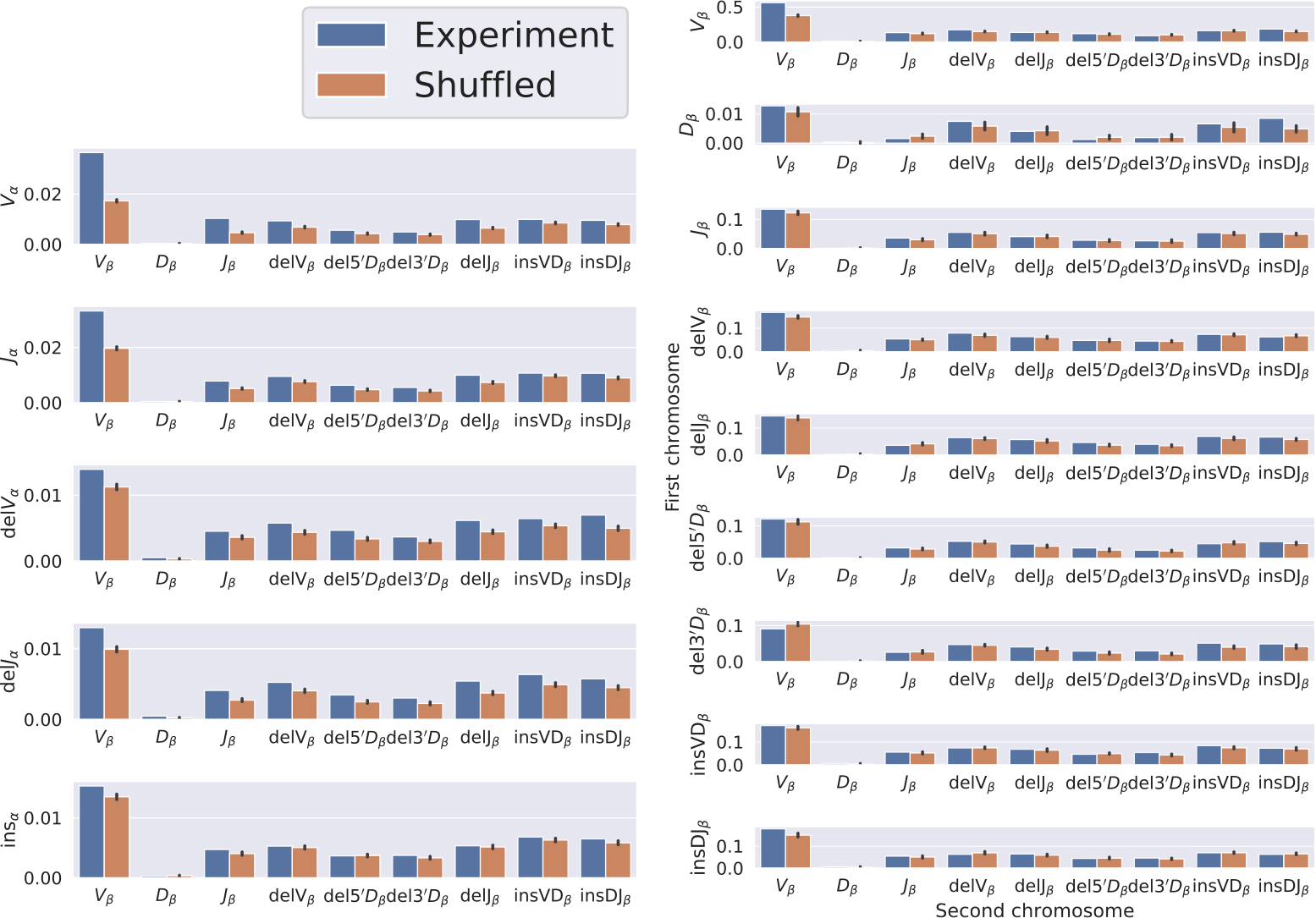

Supplement: S2 Fig — The null distribution is obtained by shuffling the pairs, the error-bar represents the standard deviation over multiple shuffling. We consider the raw mutual information, not corrected with the shuffled distribution, contrary to Fig 2. With a false discovery rate of 0.01 (using the Benjamini—Hochberg procedure) and assuming a Gaussian distribution for the mutual information of shuffled sequences, we find that, for β − β pairings, the only pairs of features passing the test are (in order of significance) V1 − V2, V1 − InsDJ2 and Del3′D1 − InsDV2. By contrast, for α − β pairing, with the same false discovery rate (0.01), 36 out of the 45 possible feature pairings are significant. (TIF) [file pcbi.1006874.s002.tif]

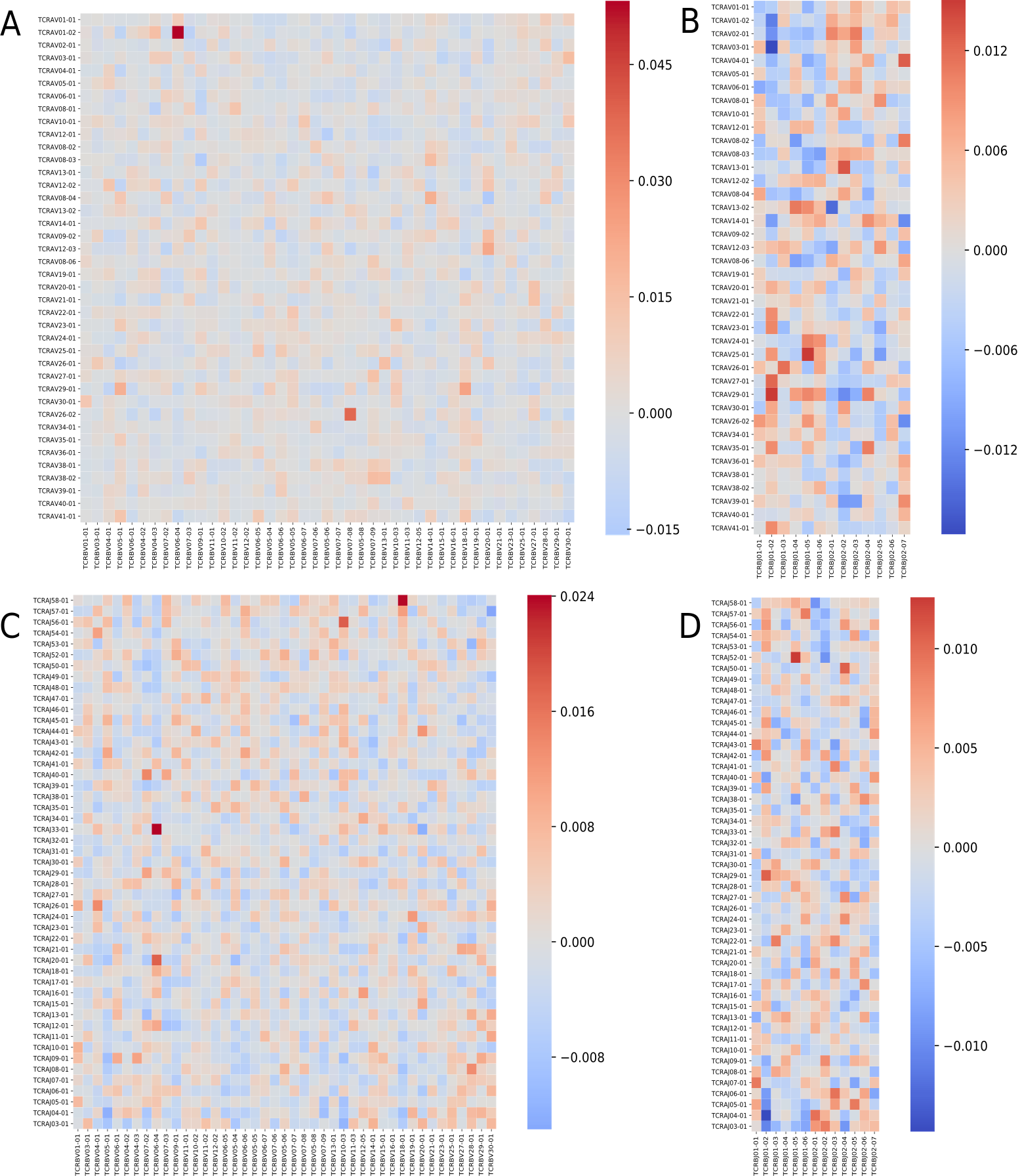

Supplement: S3 Fig — Vα − Vβ (A), Vα − Jβ (B), Jα − Vβ (C) and Jα − Jβ (D). The correlation are generically small and do not show a particular structure. (TIF) [file pcbi.1006874.s003.tif]

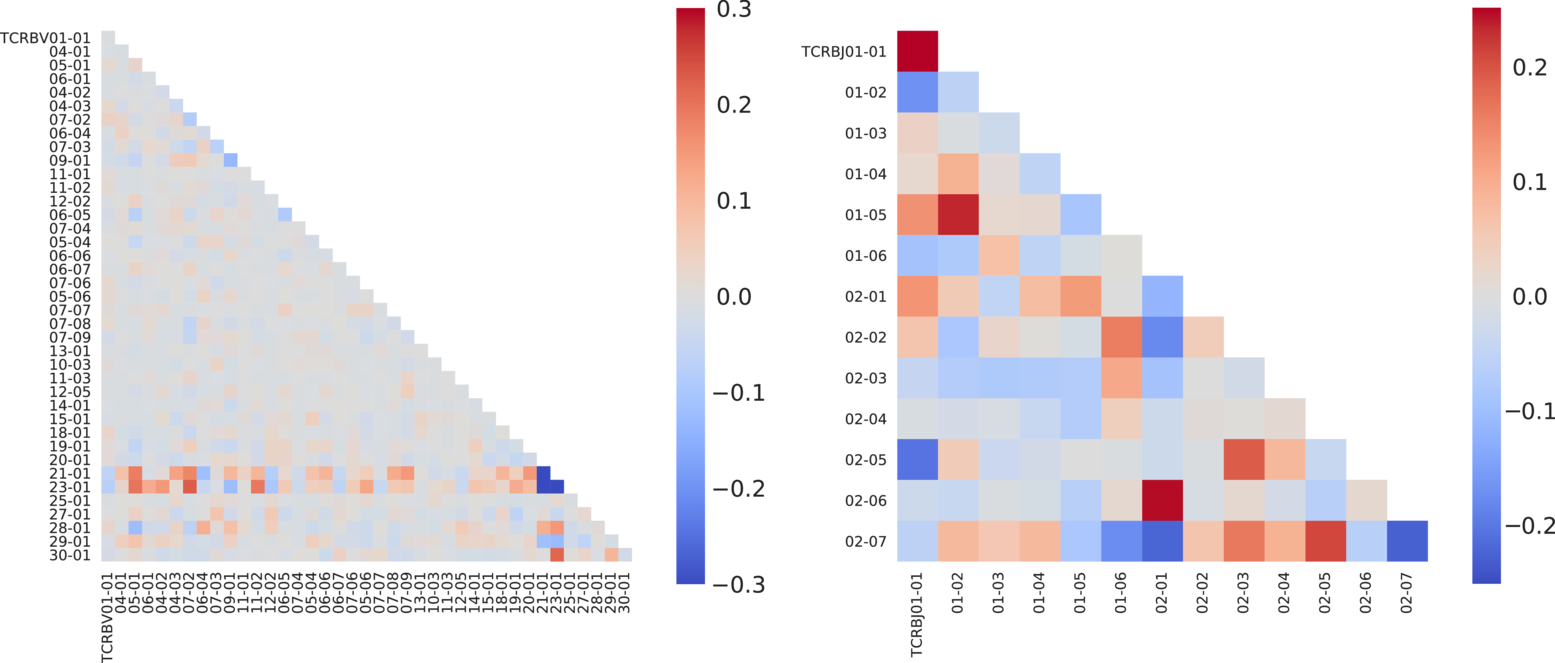

Supplement: S4 Fig — The V21-01 and V23-01 genes are non-functional pseudogenes and are thus anticorrelated. (TIF) [file pcbi.1006874.s004.tif]

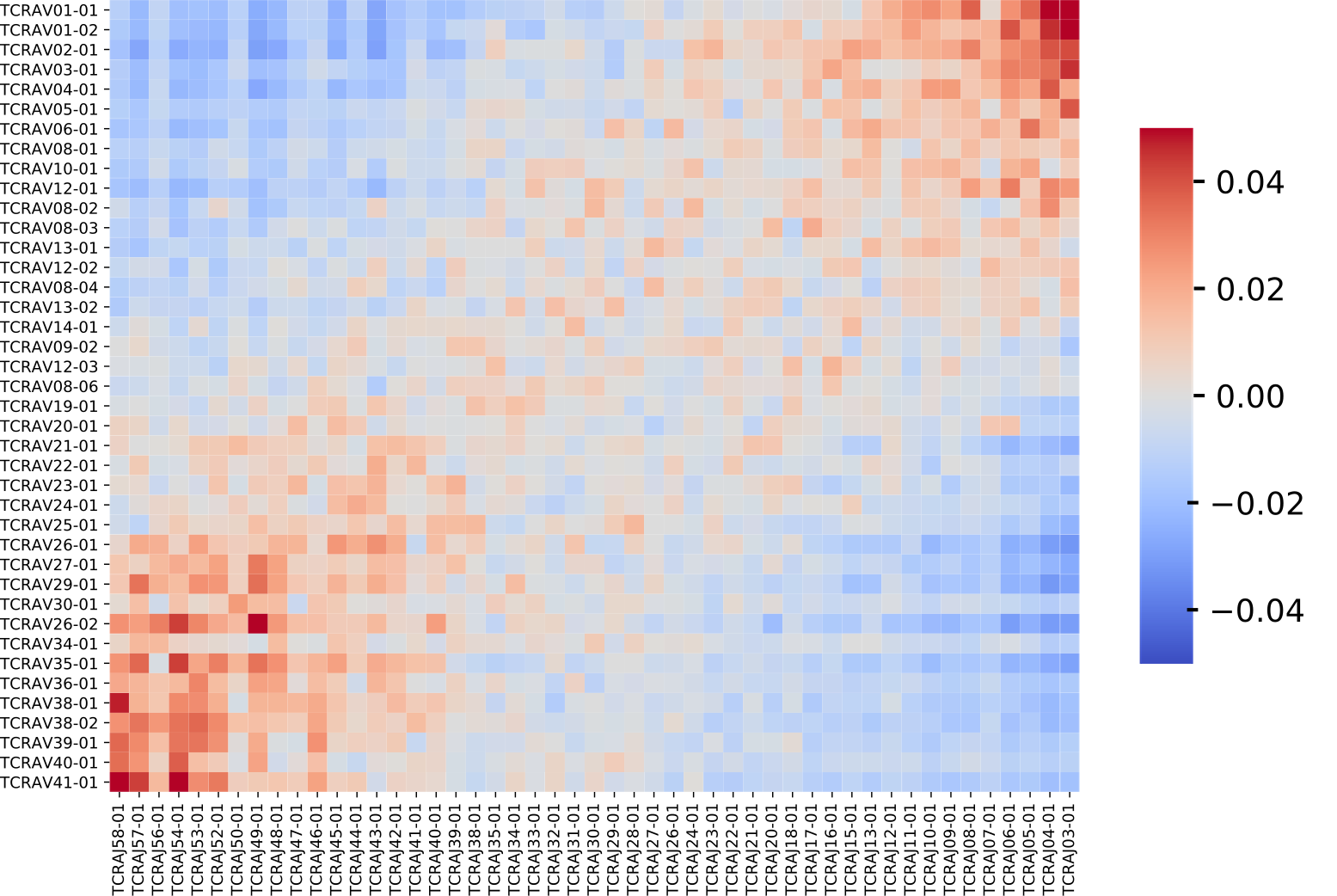

Supplement: S5 Fig — The correlations observed in Fig 3A and 3B are also observed here. (TIF) [file pcbi.1006874.s005.tif]

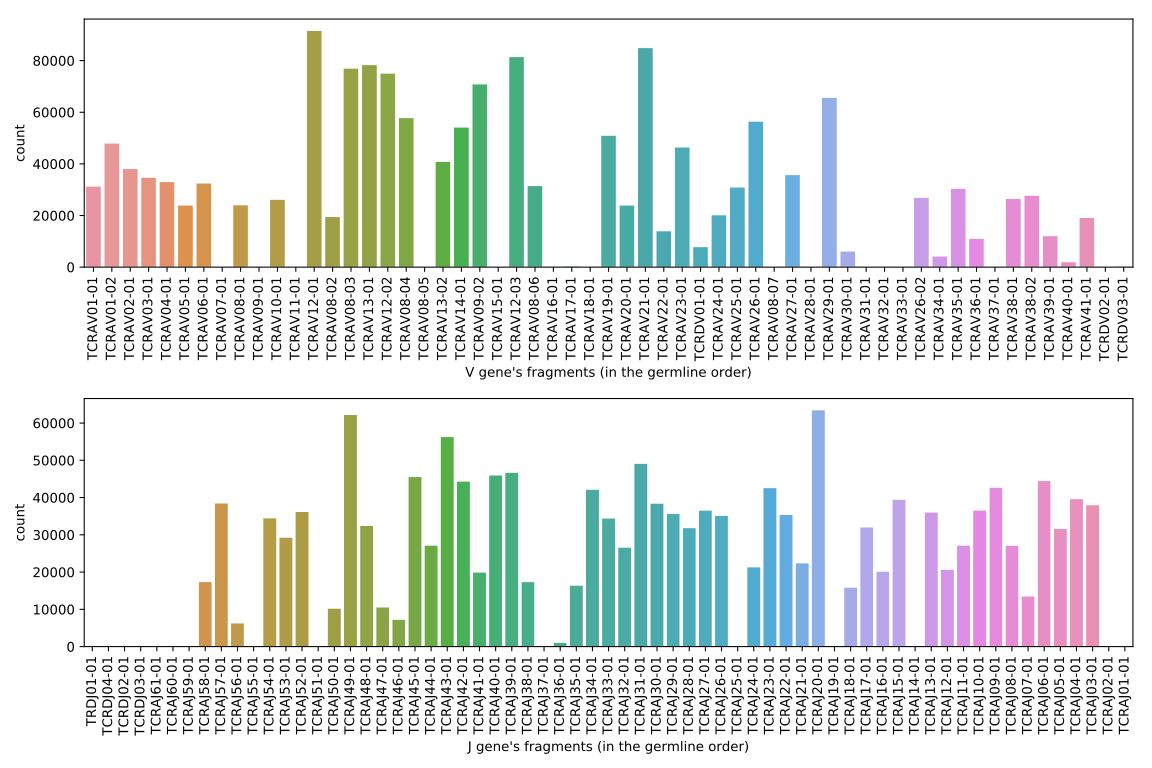

Supplement: S6 Fig — In both case, they are ordered along the germline, 5’ to 3’. (TIF) [file pcbi.1006874.s006.tif]

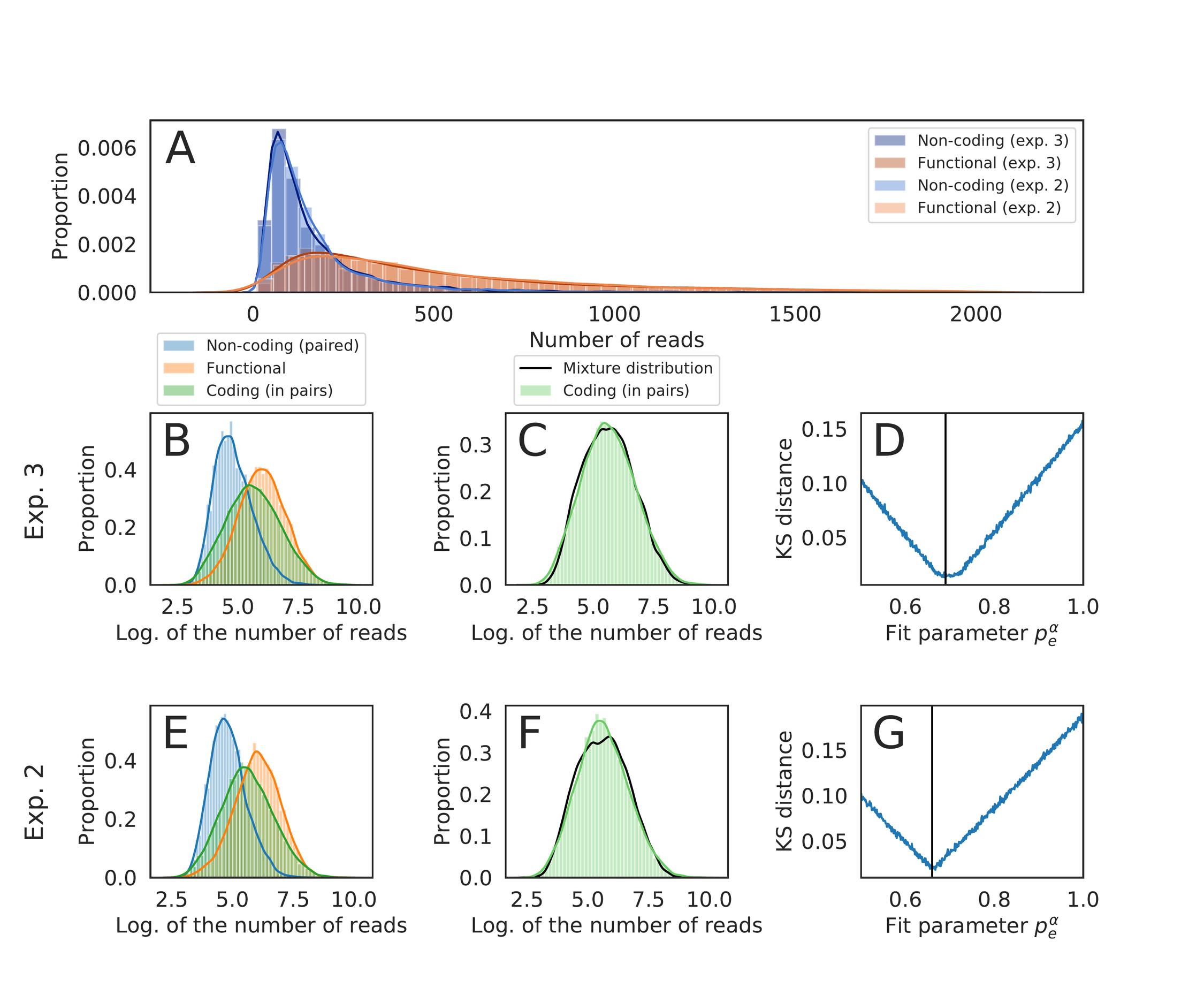

Supplement: S7 Fig — (A) displays the distribution (normalized histogram and kernel density estimation) of the total number of read counts (all wells summed) of subsets of paired TCR sequences in experiments 2 and 3. The blue histograms look only at the sequences which are paired and non-coding, while the yellow ones focus on sequences paired with a non-coding sequence, hence expected to be expressed. The histograms are normalized so that the area under them is equal to one. The bin width is chosen using the Freedman-Draconis rule. (B) (resp. (E)) shows the distribution of the log-transformed read counts for experiment 3 (resp. 2). In blue, paired non-coding sequences and in yellow functional sequences again. The green histogram corresponds to coding sequences paired with another coding sequence (CC). This last type of sequences contains both expressed and silenced sequences, the distribution of its read counts should be a mixture of the two other distributions. The parameter peα of this mixture can be related to the proportion of cells exhibiting two functional TCRα chains (see Methods). In plot (C) (exp. 3) and (F) (exp. 2), the mixture distribution, with parameter peα minimizing the Kolmogorov-Smirnov (KS) distance between the two distributions, is represented in black, while the distribution (CC) is shown in green. Plots (D) and (G) show (for experiments 3 and 2 respectively), the KS distance between the mixture distribution and the (CC) distribution for different values of the parameter peα. The fit did not depend significantly on the bin width of the histograms. The black vertical line corresponds to the value of peα giving the minimum distance, respectively 0.66 ± 0.03 and 0.69 ± 0.03 in Exp. 2 and Exp. 3. (TIF) [file pcbi.1006874.s007.tif]

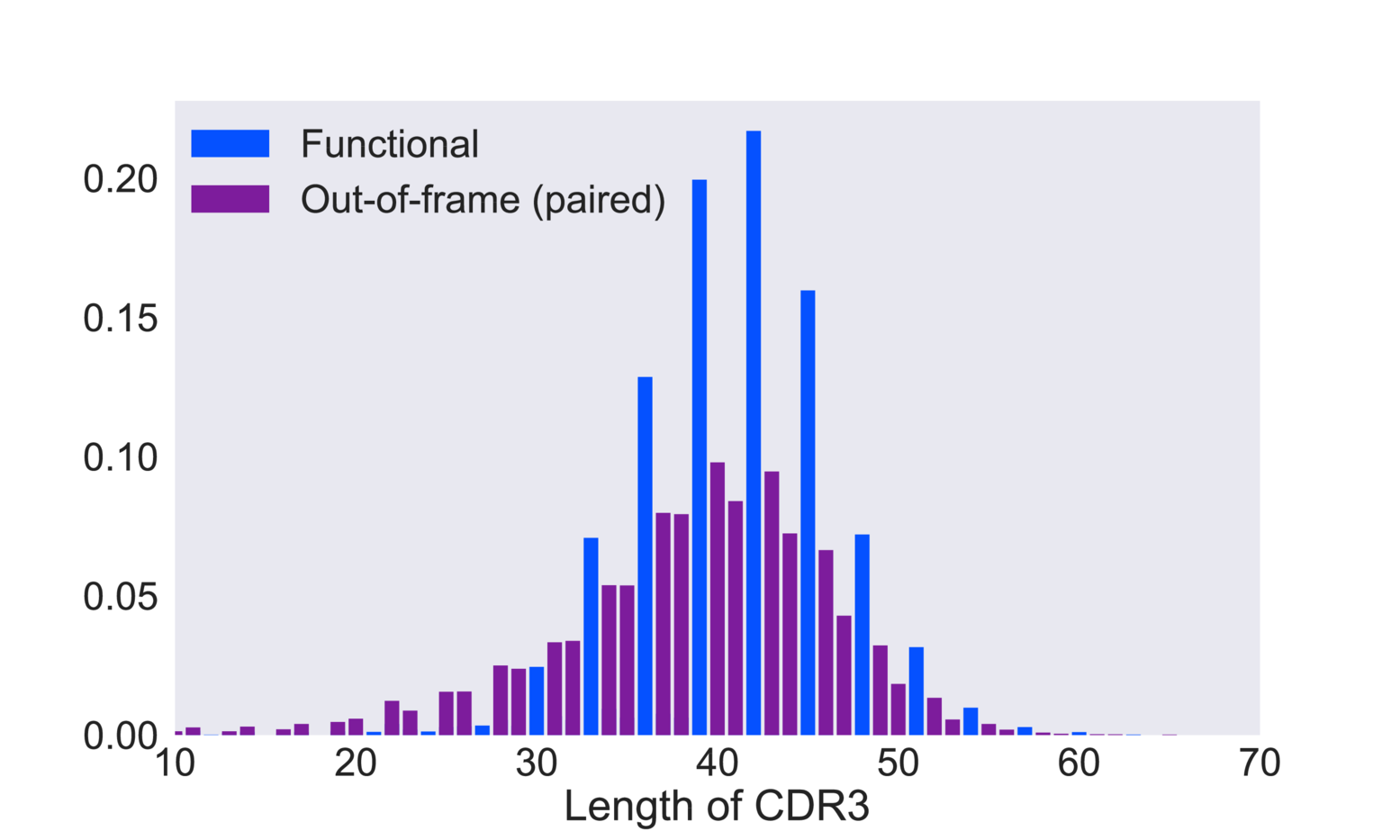

Supplement: S8 Fig — Expressed sequences have a narrowed distribution than unselected ones. All sequences used in these distributions were paired. (TIF) [file pcbi.1006874.s008.tif]

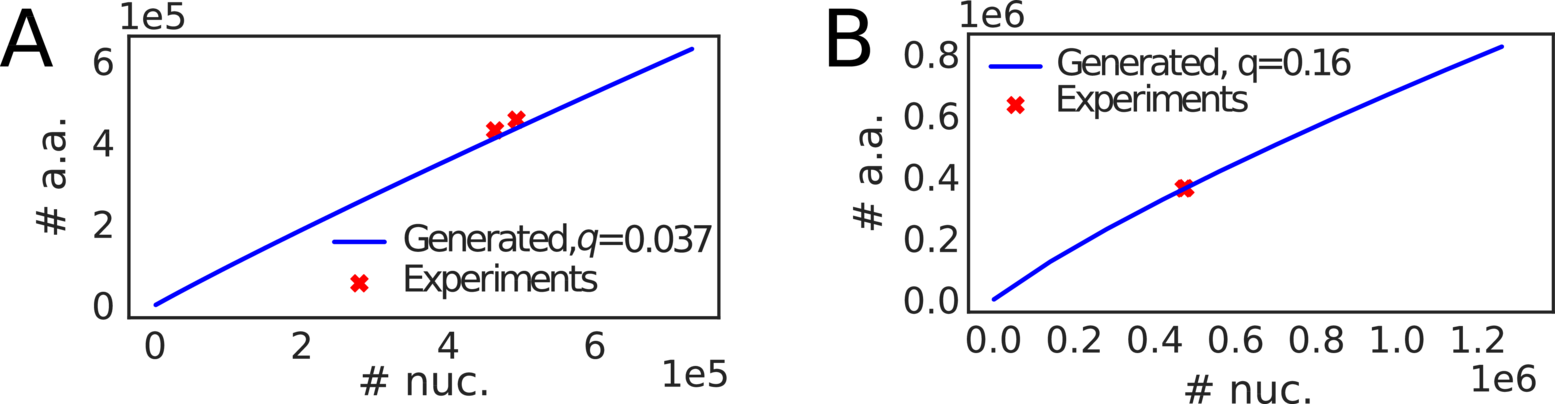

Supplement: S9 Fig — Red crosses are experimental data, blue line comes from simulations of the recombination model with random selection. For α the value of q is inferred by least-square minimisation to be qα = 0.16, while for β we used the value of qβ = 0.037 reported in Elhanati et al., Immunological Reviews, in press (2018). (TIF) [file pcbi.1006874.s009.tif]

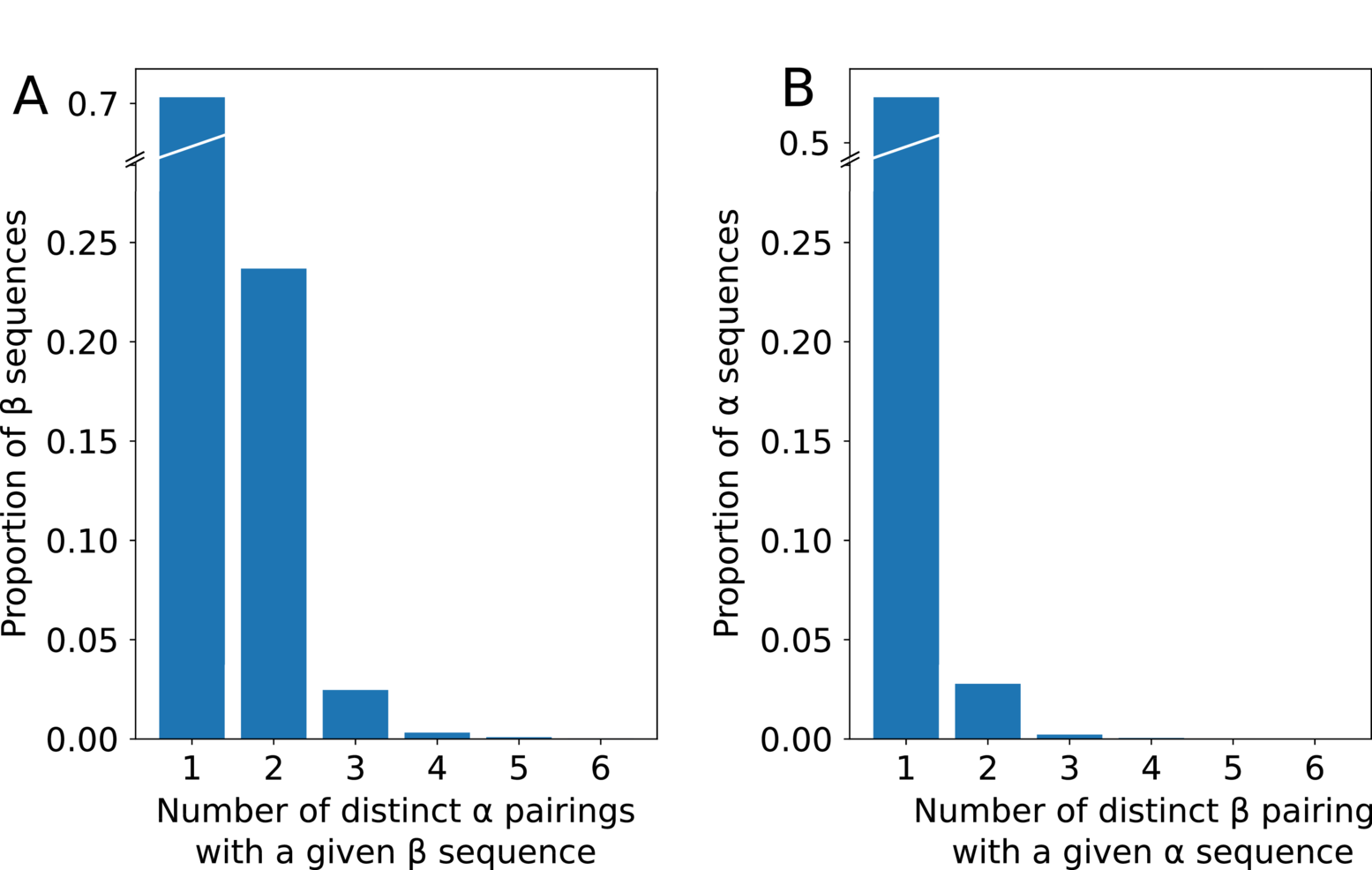

Supplement: S10 Fig — Only sequences that appear in at least a pairing are considered. Since sequences may be paired with 2 chains of the other type in a single cell, only chains with 3 or more associations unambiguously correspond to the convergent selection of that chain in different clones. (TIF) [file pcbi.1006874.s010.tif]

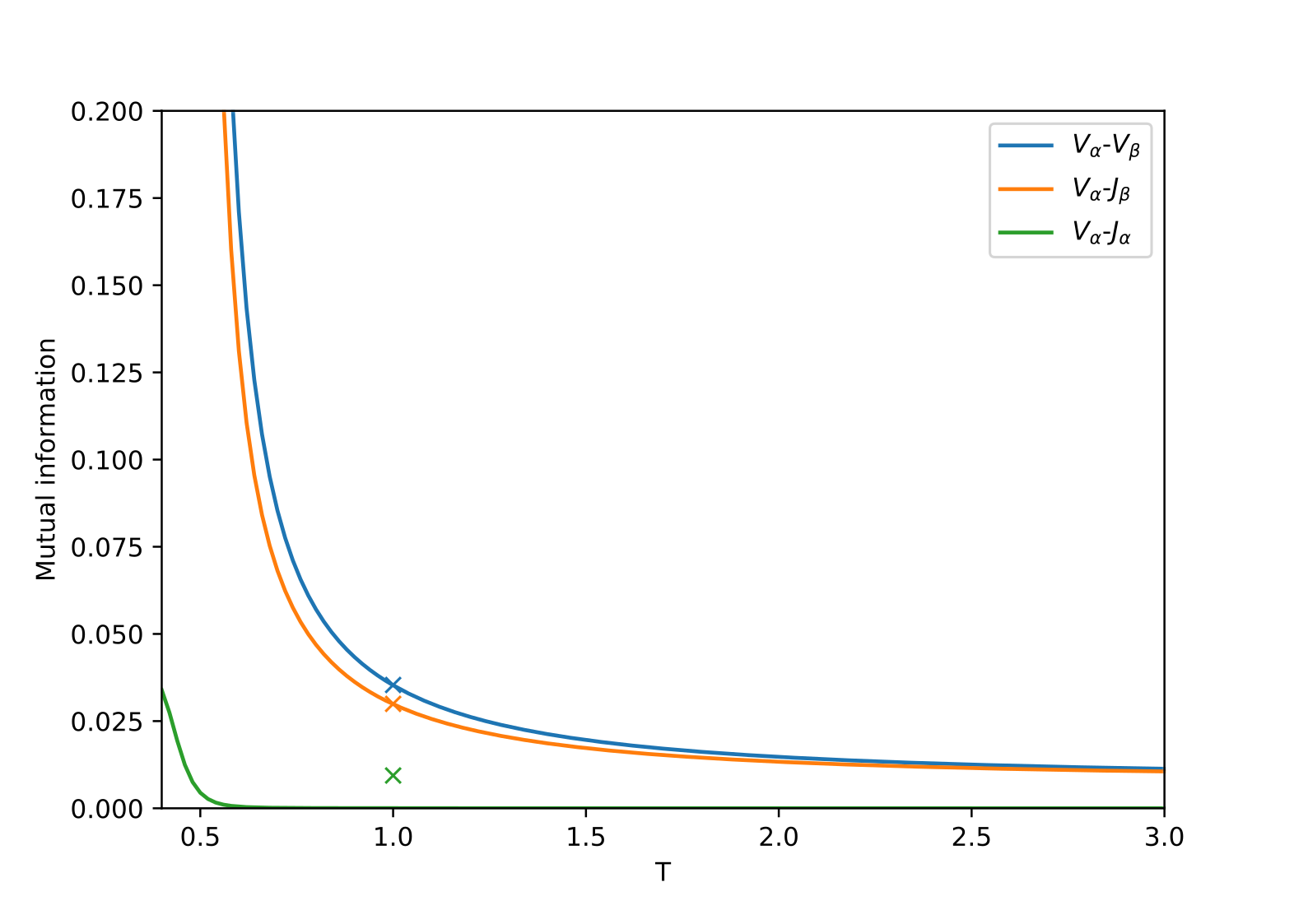

Supplement: S11 Fig — The dot are the observed values in the dataset. (TIF) [file pcbi.1006874.s011.tif]
